# Holidays

**Published:** 1900-08-25

**Authors:** 


### The Hospital

A Journal of

The Medical Sciences and Hospital Administration.

Vol. XXVIII.—No. 726.] EDITED BY SIR HENRY BURDETT, K.C.B., AND SOLOMON C. SMITH, M.D., M.R.C.P. [August 25, 1900.

### HOLIDAYS.

It has been the fortune of all sayers of wise sayings, probably from before the time of Solomon, and certainly ever since, to be continually misquoted by the majority of those who profess to share and to echo their sentiments. The late Sir William Molesworth, a somewhat cynical recluse, once expressed the opinion, when worried to join in some projected festivity, that "life would be tolerable if it were not for its amusements." Just as Solomon is supposed by the unthinking to have declared that "wisdom," instead of "safety," might be obtained from a multitude of counsellors, so Sir William's absolutely appropriate word "amusements" has been converted, by many of his professed admirers, into "pleasures," and the apophthegm, in this utterly foolish and unmeaning form, has passed from mouth to mouth with mild approval. Its whole point manifestly turns upon the implied suggestion that amusements may not be pleasures; and, from this point of view, it is probably susceptible of very wide application. One of our daily contemporaries has lately opened its columns to a discussion of the subject whether the "holidays" in which the majority of English people now think it necessary to indulge are really beneficial to those who are supposed to enjoy them; and it is at least certain that a great deal might be said on the negative side of the inquiry. Thanks to modern facilities for locomotion, the prophesy that "many shall run to and fro" has been abundantly fulfilled; but it is, perhaps, doubtful whether the succeeding clause, to the effect that "knowledge shall be increased," can be spoken of in the same manner. The sights seen and the sounds heard upon suburban roads as incidents of the conclusion of a "beanfeast," or the brakes full of weary and draggled-looking "holiday-makers" which may be encountered almost any summer evening are, at all events, suggestive of an inquiry as to how far the participators in the outing have found it possible to combine "pleasure" with "amusement."

There existed in the days of our youth, and may perhaps still be found upon bookstalls, a somewhat priggish publication entitled "Todd's Student's Manual;" and Todd, whoever he may have been, set himself to maintain that the most effective brain rest was obtained by a change of subject matter. A student weary of genders and declensions was not to walk in the fields, but was to have recourse to algebra. Todd was hardly a philosopher, and certainly was not provided with a sense of humour; but physiology lends a modified support to the doctrine which he enunciated. A change of occupation may be assumed to call a fresh set of brain-cells into activity; and this is probably better for the organ as a whole than complete torpor, except during the time which may properly be devoted to sleep. The benefits popularly supposed to be wrought by "change of air" are hardly recognisable by scientific inquiry, and it is highly probable that they are imaginary rather than real. They would arise no doubt whenever the "air" that was left behind was that of a densely crowded part of some great city, and when that which was resorted to had passed uncontaminated over moorland or ocean. Even then time would be of the essence of the implied contract with the forces of nature; and a brief holiday, however much it might be a new and pleasurable experience, would hardly increase in any appreciable degree the resisting powers of the organism. In cases which are typical of large classes, we are strongly of opinion that the annual holiday might be most profitably spent at home. Take the example of a clerk, whose stipend maintains a wholesome and comfortable little dwelling, with a few square yards of garden, within easy reach of a suburban railway station, such a dwelling as may be seen by the thousand in every district around London. His employer gives him an annual holiday, it may be of a fortnight or three weeks. Would he not spend it more wholesomely, both for himself and his family, by making it a period of increased physical rest, of somewhat earlier hours at night and of later hours in the morning, and by devoting the days to rendering his garden the envy and admiration of his neighbours, than by expending the savings of the whole year, often obtained only by considerable self-denial, on a fortnight of stuffy lodgings and general discomfort at some so-called "health resort" at the seaside, with the attendant liability to place his young children in beds recently occupied by others convalescent from measles or scarlet fever? It is trite to observe that circumstances alter cases, and no doubt there may be many in which the absence from home may be desirable. Each one must be judged upon its own merits; but it would none the less be a wholesome intellectual exercise for all recently-returned holiday-makers to take stock of the actual benefits which they have received, and to strike a balance between these and the attendant discomforts and annoyances. Such an examination would bring many to the conclusion which was reached by the late Mr. Nassau Senior, who said that, having taken thought and spent money to obtain a comfortable house of his own, he entirely declined to pass a night in any which belonged to another person.

